# High Polymorphism in the *Dmrt2a* Gene Is Incompletely Sex-Linked in Spotted Scat, *Scatophagus argus*

**DOI:** 10.3390/ani12050613

**Published:** 2022-02-28

**Authors:** Umar Farouk Mustapha, Daniel Assan, Yuan-Qing Huang, Guang-Li Li, Dong-Neng Jiang

**Affiliations:** Fisheries College of Guangdong Ocean University, Guangdong Provincial Key Laboratory of Pathogenic Biology and Epidemiology for Aquatic Economic Animals, Guangdong Province Famous Fish Reproduction and Breeding Engineering Technology Research Center, Zhanjiang 524088, China; umarfk.gh@gmail.com (U.F.M.); cobbykarsh508@gmail.com (D.A.); huangyuanqing2@stu.gdou.edu.cn (Y.-Q.H.); guangligdou@163.com (G.-L.L.)

**Keywords:** *Dmrt2a*, mutations, indels, SNPs, sex-linked, sex-chromosome, gene expression, *Scatphagus argus*

## Abstract

**Simple Summary:**

Fishes have relatively younger chromosomes than mammals and birds, making them excellent models to study sex chromosome differentiation in teleosts. The spotted scat has a young chromosome, making it an ideal model to study the evolution of sex chromosomes in vertebrates. The doublesex and Mab-3-related transcription factor 1 (*Dmrt1*) is the candidate sex determination gene in spotted scat, while the differentiation of other sex-linked genes remains unknown. *Dmrt2a* is positioned close to *Dmrt3* and farther away from *Dmrt1* on the same chromosome, while *Dmrt2b* is not. *Dmrt2a* is highly expressed in testicular tissues, with several intronic and exonic mutations that do not affect gene translation. Using intronic markers, we found in 4 different populations that *Dmrt2a* is incompletely sex-linked. Incomplete sex linkage of markers suggests the absence of recombination depression in this region and indicates that the system of sex chromosomes is still young. This suggests that *Dmrt2a* might be necessary for developmental processes. Other possibilities are that the conservation differences between *Dmrt1*, *Dmrt3,* and *Dmrt2a* might be due to gene dosage effect, or specialization of truncated copies of *Dmrt1* and *Dmrt3* in the regulation of normal gene copies. The partial sex linkage of *Dmrt2a* suggests an excellent model for studying sex-linked gene differentiation during vertebrate evolution. Therefore, further studies are needed to identify the regulators of *Dmrt2a* expression, sequences of X and Y copies, and their functions in spotted scat.

**Abstract:**

Unlike mammals and birds, many fishes have young sex chromosomes, providing excellent models to study sex chromosome differentiation at early stages. Previous studies showed that spotted scat possesses an XX-XY sex determination system. The X has a complete *Dmrt3* copy (termed normal) and a truncated copy of *Dmrt1* (called *Dmrt1b*), while the Y has the opposite (normal *Dmrt1*, which is male-specific, and a truncated *Dmrt3* called *Dmrt3△-Y*). *Dmrt1* is the candidate sex determination gene, while the differentiation of other sex-linked genes remains unknown. The spotted scat has proven to be a good model to study the evolution of sex chromosomes in vertebrates. Herein, we sequenced a neighbor gene of this family, *Dmrt2*, positioned farther from *Dmrt1* and closer to *Dmrt3* in the spotted scat, and analyzed its sequence variation and expression profiles. The physical locations of the three genes span across an estimated size of >40 kb. The open reading frames of *Dmrt2a* and its paralog *Dmrt2b* are 1578 bp and 1311 bp, encoding peptides of 525 and 436 amino acid residues, respectively. *Dmrt2a* is positioned close to *Dmrt3* but farther from *Dmrt1* on the same chromosome, while *Dmrt2b* is not. Sequence analysis revealed several mutations; insertions, and deletions (indels) on *Dmrt2a* non-coding regions and single-nucleotide polymorphisms (SNPs) on the *Dmrt2a* transcript. These indels and SNPs are sex-linked and showed high male heterogeneity but do not affect gene translation. The markers designed to span the mutation sites tested on four different populations showed varied concordance with the genetic sexes. *Dmrt2a* is transcribed solely in the gonads and gills, while *Dmrt2b* exists in the gonads, hypothalamus, gills, heart, and spleen. The *Dmrt2a* and *Dmrt2b* transcripts are profoundly expressed in the male gonads. Analyses of the transcriptome data from five other fish species (Hainan medaka (*Oryzias curvinotus*), silver sillago (*Sillago sihama*), Nile tilapia (*Oreochromis niloticus*), Hong Kong catfish (*Clarias fuscus*), and spot-fin porcupine fish (*Diodon hystrix*)) revealed testes-biased expression of *Dmrt1* in all, similar to spotted scat. Additionally, the expression of *Dmrt2a* is higher in the testes than the ovaries in spotted scat and Hainan medaka. The *Dmrt2a* transcript was not altered in the coding regions as found in *Dmrt1* and *Dmrt3* in spotted scat. This could be due to the functional importance of *Dmrt2a* in development. Another possibility is that because *Dmrt2a* is positioned farther from *Dmrt1* and the chromosome is still young, meaning it is only a matter of time before it differentiates. This study undeniably will aid in understanding the functional divergence of the sex-linked genes in fish.

## 1. Introduction

The evolution of sex determination (SD) genes, sex chromosomes, and SD systems have attracted the interest of biologists for decades. The sex determination systems of most mammals and birds are XX-XY and ZZ-ZW, and the Y/W chromosome is degraded in varying degrees [1]. Over 30,000 known fish species have genetic sex determination, environmental sex determination, and combined environmental and genetic sex determination systems [2]. The genetic sex determination of fishes includes XX-XY, ZZ-ZW, and multiple sex chromosomes. Most fish sex chromosomes are poorly differentiated, making it challenging to identify sex chromosomes and sex-determining genes. On the other hand, with the assistance of modern molecular and sequencing technologies, many SD and candidate SD genes have been cloned. In 2002, the first fish sex-determining gene, the DW domain gene on the Y chromosome (*dmy*), a duplicated copy of *dmrt1* in the sex-determining region of the Y chromosome (*dmrt1bY*), was cloned in Japanese medaka (*Oryzias latipes*). Sex-determining and candidate genes were cloned in only around ten fish species [3,4,5,6]. The origins of new sex determination genes fall into two categories, including allelic diversification and gene duplication followed by neo- or sub-functionalization [7]. Sex chromosome is originated from the autosome. Firstly, a novel sex-determining gene is evolved on the autosome. Some sexually antagonistic alleles (genes beneficial to one sex but harmful to the other) evolve near the sex-determining gene. The number of sex antagonistic genes continues to increase, and the region of sex determination accumulates repetitive sequences. The recombination of sex chromosomes inhibits the formation of a sex-linked differentiation region, and the sex chromosomes gradually differentiate [8]. Most fish lack differentiated sex chromosomes, and the sexes of many fish species are controlled by genetic factors on the young sex chromosomes, making fishes good models for studying the early formation of sex-determining genes and sex chromosomes in vertebrates. The presence of sex-determining genes that should not recombine and the evolution of Y-linked genes that benefit only male functions can lead to recombination suppression between sex chromosomes. Recombination between genes is affected by evolution, with genes in which recombination stopped before the splitting of taxa being less diverged than those after [9], indicating a relationship between age and recombination between genes.

The doublesex and mab-3-related transcription (Dmrt) member genes are intensely conserved regulators in the sexual development of metazoan [10]. The Dmrt genes have been reported in more than 30 fish species, most of which are related to sexual development [11]. The Dmrt member genes widely encode proteins with highly conserved DM domain and exist differently in both vertebrates and invertebrates, suggesting the possibility of additional functions besides sexual development [12,13,14,15,16]. The genes encompass major transcription factors involved in neurogenesis, sex determination and differentiation pathways, organ development, growth and maintenance, and somite differentiation [17,18,19]. In humans, 8 Dmrt member genes exist, along with 7 in mice, 11 in *C. elegans*, 4 in *Drosophila*, and 6 in fish (*Dmrt1–6*) [3,11,12,13,14,15,20,21,22,23,24,25]. The fish *Dmrt1–Dmrt5* genes were relatively conserved while *Dmrt6* was lost in most species during the evolutionary process [11]. However, in mice, multiples of *Dmrts* have been reported to participate in sexual development [21]. Similarly, duplicates of *Dmrt1*, -*2*, and -*3* have been identified in most fish species [3,12,13,26,27].

*Dmrt2* is an important Dmrt member gene but is controversial due to its participation in gonad development and other non-gonadal functions [19,28]. The *Dmrt2* is essential for somite development and left–right patterning, and is expressed in the dermomyotome of developing vertebrate somites [29,30]. In situ hybridization revealed that *Dmrt2a* is expressed in germ cells of the developing swamp eel [31]. In *Megalobrama amblycephala*, *Dmrt2a* and *Dmrt2b* essentially showed similar expression levels between testis and ovary samples [13]. However, in species such as Medaka, *Oryzias latipes* [22], zebrafish, *Danio rerio* [26], swamp eel, *Monopterus albus* [31], and blunt snout bream *Megalobrama amblycephala* [13], *Dmrt2/Dmrt2a* was more predominantly expressed in the testicular tissues than in the ovaries.

Spotted scat is an important marine culturable fish distributed in Southern and Eastern Asia. Its moderate body size and flat shape make it a good experimental fish. Sequence analysis and the development of sex-linked markers indicate that *Dmrt1* is tightly linked to male sex in spotted scat [3]. Additionally, genome sequencing revealed that *Dmrt1* only existed in XY male fish, while the mutated copy of *Dmrt1* (*Dmrt1b*) is located on the X chromosome [32,33]. The male-specific *Dmrt1* is the candidate SD gene in spotted scat [3]. The *Dmrt3△-Y* is a truncated mutation of *Dmrt3*, and it’s also Y-linked in spotted scat. On the other hand, the situation for *Dmrt2a* is not known in spotted scat. In this study, we sought to determine whether the *Dmrt2a* is sex-linked, and if so, whether sex linkage is complete and whether the Y- and X-linked copies differ in sequence or expression. Herein, we isolated *Dmrt2a* and *Dmrt2b* from spotted scat. The genomic organization revealed a size variation of insertions and deletions (indels) of *Dmrt2a*. The indels are sex-linked at different rates across populations, while the expression of *Dmrt2a* in the gonads of spotted scat is in line with previous reports.

## 2. Materials and Methods

### 2.1. Animals and Sample Collection

Initially, adults *Scatophagus argus* was purchased from Dongfeng Market (Zhangjiang, Guangdong, China) and transported to the laboratory with adequate oxygen. Fish weights and measurements were taken and sacrificed by decapitation after 100 mg/L of tricaine methanesulfonate (MS 222, Sigma, Saint Louis, MO, USA) anesthetization. The average lengths and weights of the adult spotted scat used in this study were 18.5 cm and 169.9 g for male and 19.2 cm and 204.5 g for female, respectively. Male and female tissues, including hypothalamus, pituitary, gonad, gill, heart, kidney, liver, spleen, stomach, intestine, and muscle tissues, were carefully excised and snap-frozen in liquid nitrogen, then stored at −80 °C before RNA isolation. Tail fins were also taken for DNA extraction. Parts of the gonadal samples were kept in Bouin’s solution for further histological studies (hematoxylin and eosin staining). For further analysis, we sampled fish from four different populations, from Donghai Dao, Beihai, Xiashan, and Zhuhai. The population from Donghai Dao was artificially bred from mixed parents, while the population from Zhuhai was bred from two parents and twenty-four progenies. The populations from Beihai and Xiashan were obtained from the wild with unknown genetic background. All experimental fish protocols followed the guidelines and approval of the Administration of Affairs Concerning Experimental Animals for the Science and Technology Bureau of China, and the Animal Research and Ethics Committees of Guangdong Ocean University.

### 2.2. RNA Isolation and cDNA Synthesis

Total RNA was prepared using TRIzol reagent (Invitrogen), according to the manufacturer’s instructions. The RNA integrity was assessed on 1% agarose gel electrophorese and visualized by ethidium bromide. Approximately 1 μg of total RNA from each sample was used for the first-strand cDNA synthesis using the PrimeScript™ RT reagent kit with gDNA Eraser (Takara, China), according to the manufacturer’s instruction.

### 2.3. Genotypic and Phenotypic Sex Identification

Nucleic acid purification kit (N1173, Dongsheng Biotecon, Guangzhou, China) was used for the DNA extraction following the manufacturer’s instruction. Sex-specific DNA Marker (Dmrt1-Marker-F/R) (forward primers: 5′- GAAGGCAGCA AGATCAGGAGGA-3′ and reverse primers: 5′- CAGCAGCAGGTCAGATGGTTCC-3′) as described in [3,34] was used to determine the genetic sex of the spotted scat. Hematoxylin and eosin staining (H&E) of spotted scat gonads was also employed to confirm the genotypic sex and gonad developmental stage determination described in our previous studies [3,34]. The Dmrt1-Marker-F/R used for the genetic sex identification was previously found to be 100% accurate when tested on 113 males and 133 females from five different populations (both wild and hatchery) in China [3]. The current study also observed a 100% concordance rate of the Dmrt1-Marker-F/R with the phenotypic sex as determined by H&E staining.

### 2.4. Cloning of Dmrt2a and Dmrt2b in S. argus

The complete cDNA sequences of *S. argus Dmrt2a* and *Dmrt2b* were obtained from our mixed tissues [35] and gonad [36] transcriptome data. Nile tilapia *Dmrt2a* (NCBI accession number: NP_001266696) and *Dmrt2b* (NCBI accession number: AAX08123) mRNA sequences were used as query sequences to blast our transcriptome sequence data.

Primers flanking the open reading frame (ORF) were designed with reference to the transcript obtained, and the full lengths of *S. argus Dmrt2a* and *Dmrt2b* were cloned as described in [3]. The PCR protocol used was as follows: initial denaturation at 94 °C for 3 min, 37 cycles of denaturation at 94 °C for 30 s, annealing at 60 °C for 30 s and extension at 72 °C for 2 min, followed by a final extension of 10 min at 72 °C. The PCR products were then examined on 2% agarose gel stained with ethidium bromide. Amplification bands were extracted from the gel, purified, and cloned into the p-Easy-T3 vector (TransGen Biotech, Beijing, China) as described previously [34]. All primers used and the GenBank accession numbers of Dmrt genes are listed in Tables S1 and S2, respectively.

### 2.5. Sequence Analysis and Bioinformatics

The cDNA sequences of *Dmrt2a* and *Dmrt2b* were spliced and analyzed by DNASTAR software (http://www.dnastar.com/, accessed on 2 October 2021). ORFs and protein sequences were predicted using the NCBI ORFfinder software (https://www.ncbi.nlm.nih.gov/, accessed on 15 October 2021). Nucleotide and protein sequence homology and multiple alignments were performed using MegAlign in the DNASTAR software and ClustalX (http://www.clustal.org/, accessed on 17-10-2021), respectively. The neighbor-joining method was used to construct the phylogenetic tree in MEGA 6.06 (http://www.megasoftware.net/, accessed on 17 October 2021) with a bootstrap value of 1000 replicates. The conserved regions of the putative amino acids sequences were predicted using SMART (http://smart.embl-heidelberg.de/ accessed on 17-10-2021). The protein sequences of *Dmrt2a* and *Dmrt2b* in other fish were obtained from the NCBI database and Ensembl Genome Browser (http://www.ensembl.org/Multi/Tools/Blast?db=core, accessed on 20 October 2021), and their accession numbers are listed in Table S2, including the spotted scat *Dmrt2a* and *Dmrt2b* cloned in this study.

### 2.6. Designing of Markers on Dmrt2a Genomic DNA Sequence

Based on the assembly of gDNA sequence from male and female spotted scat, markers were developed on sections of the sequence with insertions and deletions (indels). Five insertions and deletions were found, and five markers were designed. The marker with long indels (99 bp) was analyzed on agarose gel electrophoresis, while those with short indels (8 bp–14 bp) were analyzed on PAGE gel. According to the indel position, the markers spanning indel1, indel2, indel3, indel4, indel5, and del6 were named marker-1, marker-2, marker-3, marker-4, marker-5, and marker-6, respectively (Table 1), whereas marker-7 spanned across three SNPs (I, II, and III).

### 2.7. Tissue Distribution

Tissue distribution analysis was performed by reverse-transcription PCR (RT-PCR). Here, *β-actin* (accession number: KC161966) expression was used as an internal reference for gene normalization. The *Dmrt2a*, *Dmrt2b*, and *β-actin* primers for tissue distribution are listed in Table S1. The amplification regime consisted of 35 cycles (*β-actin* 28 cycles) of 30 s at 95 °C, 30 s at 60 °C, and 30 s at 72 °C; followed by further extension at 72 °C for 10 min. PCR products were separated on a 2.0% agarose gel and visualized with ethidium bromide.

### 2.8. Real-Time Quantitative PCR (qPCR)

Ovaries and testes were dissected from female and male adult fish. Total RNA extraction and cDNA syntheses were carried out as described previously [37]. The relative levels abundance of *Dmrt2a* and *Dmrt2b* transcripts were evaluated using the formula (R = 2^−∆∆Ct^). Here, *β-actin* was used as a reference gene to normalize the expression values. The qPCR primer sequences are listed in Table 1. Data are presented as the means ± SD. Statistical analysis was performed using SPSS 16.0 (SPSS, Chicago, IL, USA). Significant differences between groups were analyzed via one-way ANOVA with Duncan’s post-hoc test using a confidence level of *p* < 0.05.

### 2.9. Dmrt2a and Dmrt2b Expression in Transcriptome Data of Different Fish Species

We analyzed various transcriptome data for gonadal tissues to understand the expression patterns of the *Dmrt2a*&*b* in different species. Either zebrafish or spotted scat *Dmrt1*, *Dmrt2a*, and *Dmrt2b* transcripts were used as query sequences to blast the transcriptome data (cds) of spotted scat [36], Hainan medaka (*Oryzias curvinotus*) [38], silver sillago (*Sillago sihama*) [39], Nile tilapia (*Oreochromis niloticus*) [40], Hong Kong catfish (*Clarias fuscus*) [41], and spot-fin porcupine fish (*Diodon hystrix*) [42]. For confirmation, the results with different transcript numbers and maximum hits were blasted using NCBI nucleotide blast (https://blast.ncbi.nlm.nih.gov/Blast.cgi, accessed on 15 October 2021). The transcript number of confirmed genes from the cds was traced into the annotations to identify the expression in either fragment per kilobase per million mapped read (FPKM) or reads per kilobase per million mapped read (RPKM) values.

## 3. Results

### 3.1. Cloning and Sequence Analysis of Spotted Scat Dmrt2a and Dmrt2b

**Figure 1:**
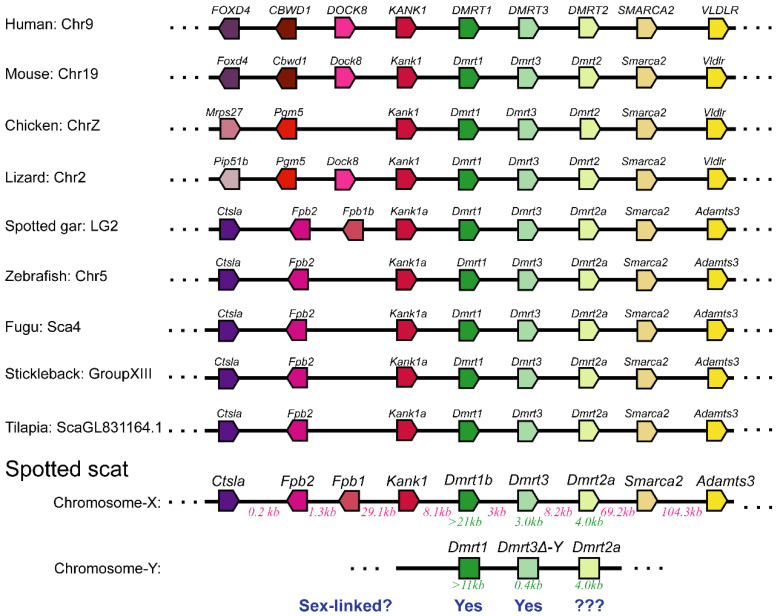
Syntenic analyses of *Dmrt1* and *Dmrt3* and their adjacent genes in different species. The figure was adopted with modifications from [3]. The bar lengths are disproportionate to the distances between genes. *Dmrt1* and its downstream gene *Dmrt3△-Y* are sex-linked in spotted scat, whiles *Dmrt2a* is unknown. The distance between spotted scat Dmrt genes is shown in red (in kb). The lengths of Dmrt genes are shown in green (in kb). The neighbor genes of Dmrts on the X-chromosome were obtained from our previous XX genomic data (https://ngdc.cncb.ac.cn/search/?dbId=gwh&q=GWHAOSK00000000.1, accessed on 5 February 2022).

Previously, we showed that the spotted scat *Dmrt1* is a candidate sex determination gene. In spotted scat *Dmrt1* is an upstream gene of *Dmrt3△-Y* [3], while *Dmrt2a* is a downstream gene of *Dmrt3△-Y*. Previously, *Dmrt1* and *Dmrt3△-Y* were found to be sex-linked, while *Dmrt2a* was not known, but detected to be sex-linked in this study (Figure 1). The neighbor genes of *Dmrt1b* and *Dmrt2* on spotted scat X-chromosome are similar to other species, whiles that of spotted scat is not yet clear (Figure 1).

The estimated length of *Dmrt1* (Exons 1, 2, 3, 4, 5 and Introns 1, 2, and 3) excluding the length of intron 4 is >11 kb, while *Dmrt1b* is >21 kb. The distance between *Dmrt1*, *Dmrt3△-Y*, and *Dmrt2a* is unclear and will be elucidated. The estimated distance between *Dmrt1b and Dmrt2a* is 14.2 kb, while *Dmrt1 and Dmrt2a* may also be similar. The physical location of the three genes spans across an estimated size of 40 kb.

**Figure 2:**
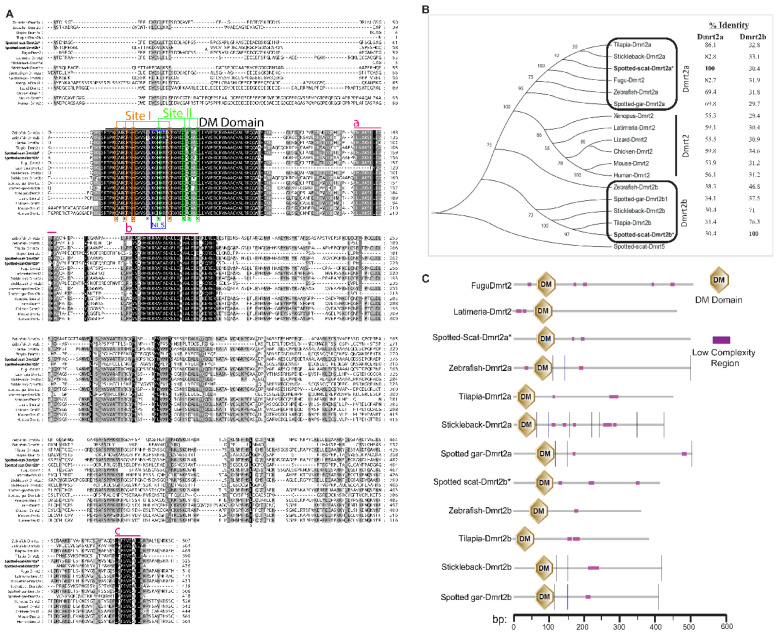
Homologous comparison of spotted scat *Dmrt2a* and *Dmrt2b* amino acid (aa) sequences with other species. (**A**) Alignment of *Dmrt2a* and *Dmrt2b* (aa) from different species. Amino acid (aa) sequences are numbered to the right. The dark overline indicates the conserved DM domain region. The two intertwined zinc-binding sites, site I (CCHC) and II (HCCC), are shown. The putative nuclear localization signal NLS is indicated. Red over lines numbered a, b, and c are other *Dmrt2* conserved regions. Asterisk indicates the conserved cysteine and histidine residues. (**B**) Phylogenetic analysis of *Dmrt2* proteins from different species based on outgroup of spotted scat Dmrt5. The neighbor-joining method of MEGA 6 was used to develop the tree. Numbers on nodes indicate the credibility of the branches using 1000 bootstrap replicates. Asterisks indicate cloned spotted scat *Dmrt2a* and *Dmrt2b* and clustered among their counterparts. The identities of spotted scat *Dmrt2a* and *Dmrt2b* amino acid sequences with other spices are shown on the right side. (**C**) Schematic representation of *Dmrt2* domain features of different species predicted by SMART (http://smart.embl-heidelberg.de/, accessed on 17 October 2021). The scale bar indicates the length of amino acids (aa) in base pairs (bp). The hexagons indicate DM domain regions. The accession numbers of the sequences used are described in Table S2.

The lengths of spotted scat *Dmrt2a* and *Dmrt2b* genes isolated in this study were 1578 and 1311 base pairs (bp) encoding proteins of 525 and 436 amino acid (aa) residues, respectively (Figure S1A,B). Sequence alignment indicated that *Dmrt2a* and *Dmrt2b* have important DM domains, while the cysteine and histidine residues were well conserved in all species analyzed (Figure 2A and Figure S1). Additionally, located within the DM domain region of *Dmrt2a* and *Dmrt2b* is the conserved nuclear localization signal (NLS) sequence (KGHKK/R) and two intertwined zinc-binding sites (thus, sites I and II) for (*Dmrt2a*: C72/C75/H78/C91 and H87/C96/C98/C101) and (*Dmrt2b*: C69/C72/H75/C88 and H84/C93/C95/C98), respectively (Figure 2A). Little homology was observed outside the DM domain region in all species.

Based on the phylogenetic analysis, *Dmrt2*s analyzed were grouped into three clusters (*Dmrt2a*, *Dmrt2*, and *Dmrt2b*). The overall sequences of spotted scat *Dmrt2a* and *Dmrt2b* share 30.4% identity. Phylogenetically, spotted scat *Dmrt2a* and *Dmrt2b* are much closer to their counterparts in tilapia, sharing the highest percentage identity (86.1% and 76.3%, respectively). Comparatively, all teleosts *Dmrt2a* and *Dmrt2b* were clustered separately into different clades, indicating similarities and dissimilarities (Figure 2B). Additionally, SMART blast analysis predicted the spotted scat *Dmrt2a* and *Dmrt2b* to be similar to their fish counterparts (Figure 2C).

### 3.2. The Dmrt2a Genomic Sequence in Female and Male Spotted Scat Is Dissimilar

**Figure 3:**
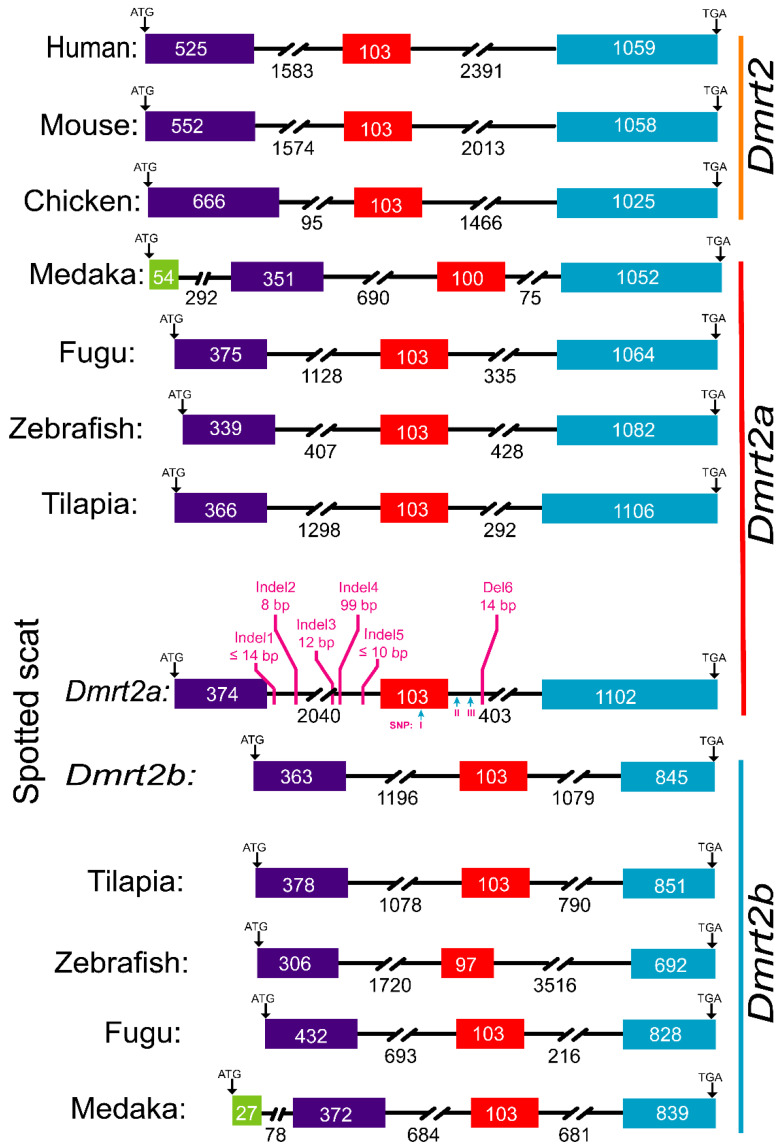
Schematic representation of *Dmrt2a* and *Dmrt2b* genes from different species. Boxes and lines indicate exons and introns, respectively. The same colors indicate the same exons. The lengths in bp (base pair) of exons are shown in the boxes, while introns are shown below. Downward arrows indicate start (ATG) and stop (TGA/TAA) codons. Red vertically inclined lines indicate insertions and deletions (indels) on spotted scat *Dmrt2a.* The polymorphisms in *Dmrt2a* were obtained from the differences in male and female sequences.

Genotypic and phenotypic sex identification of spotted scat was carried out as described in [3] before subsequent use for other analysis. The initial analysis of the genomic sequence of male and female spotted scat revealed some dissimilarities. The information regarding the genomic sequence data was previously published by our group [32]. Further gDNA cloning and sequence analysis from 3 males and 3 females revealed dispersed insertions and deletions (indels) in introns 1 and 2 (Figure 3). However, the gene structures of spotted scat *Dmrt2a* and *Dmrt2b* are comparable to other species, except that the intron 1 of *Dmrt2a* is more extended (2040 bp) than that of other species, with the closest being zebrafish with 1720 bp. The vast difference between the intron 1 of spotted scat and other species is probably due to the numerous indels (Figure 3). In addition, three SNPs, one on exon 2 (I) and two on intron 2 (II and III), were also found. However, the SNP I on the coding region did not change the amino acid sequence, meaning it could not result in functional mutation.

### 3.3. The Interspersed Indels and SNPs on Dmrt2a Are Sex-Linked at Different Rates

**Figure 4:**
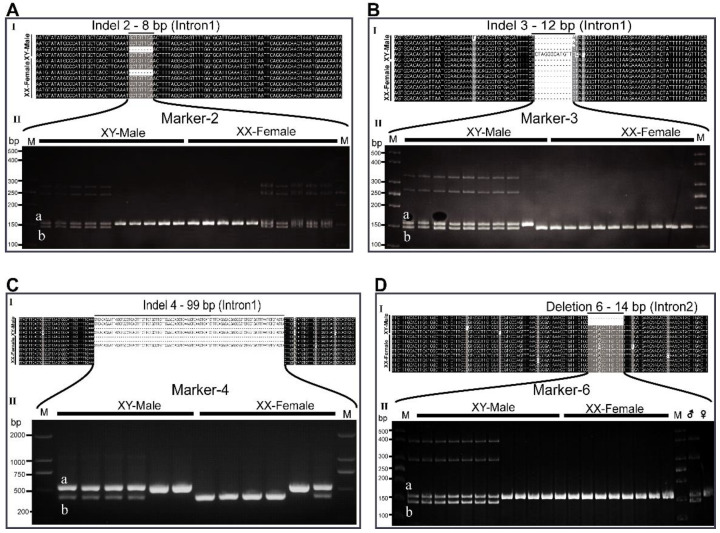
*Dmrt2a* sequence analysis in spotted scat. (**AI**) A 8 bp indel in intron 1 of male and female sequence (**AII**) PAGE gel with primers spanning the indel region was heterozygous (double bands) and homozygous (single bands) in both males and females. (**BI**) A 12 bp indel in intron 1 of the male sequence. (**BII**) PAGE gel shows that most males are heterozygous (double bands), while all females are homozygous (single bands). (**CI**) A 99 bp indel in intron 1 of male and female sequences (**CII**) Agarose gel electrophoresis reveals heterozygosity and homozygosity in males and females. (**DI**) A 6 bp deletion in intron 2 of the male sequence. (**DII**) PAGE gel shows that most males are heterozygous while all females are homozygous. The total number of fish tested with these markers is shown in Table 1 and Table 2. a; upper/longer bands, b; lower/shorter bands.

Seven primer pairs (markers) flanking the indels, including one on the SNP region, were designed (Figure 3, Table S1) and initially tested on a few individuals (Figure 4 and Figure S2). Marker-1 and marker-5 span indel1 and indel5, respectively, as well as amplified bands that are not distinguishable between males and females. The indels contained in marker-1 and marker-5 are preceded by simple sequence repeats (SSR) of at least 16 TG repeats. Interestingly, indel1 and indel5 share similar features and are the first and last indels on intron 1, respectively (Figure 3 and Figure S2).

On the contrary, primers pairs of marker-2, marker-3, marker-4, and marker-6 spanning indel2, indel3, indel4, and del6, respectively, are partially sex-linked (Figure 4). Indel2, indel3, and indel4 are 8 bp, 12 bp, and 99 bp long, respectively, on intron 1, while del6 is 14 bp long on intron 2 (Figure 4A–D). Marker-2, marker-3, marker-4, and marker-6 showed double bands in most males and single bands in most females. The upper and lower fragments are as follows: marker-2 (a, 147 bp; b, 139 bp); marker-3 (a, 160 bp; b, 148 bp); marker-4 (a, 543 bp; b, 444 bp); marker-6 (a, 158 bp; b, 144 bp). The different intensity levels of the lower and upper bands of marker-4 could be due to differences in copy number or the lengths of the two fragments. The upper bands of marker-2 and marker-6 are found in all male and female individuals. The lower bands of marker-2 are also present in some males and females, while the lower bands (b 144 bp) of marker-6 are restricted to some males. Similarly, the upper band of marker-3 (a 160 bp) is restricted solely to most males, with the lower (b, 148 bp) band present in all females (Figure 4). 

Further analyses of the markers in the four populations indicated different concordance rates (Table 1 and Table 2). Marker-1 and marker-5 were not distinguishable by PAGE gel (Figure S2). Marker-2 and marker-4 amplified double and single bands in some males and females from the four different populations. Marker-3 and marker-6 consistently amplified only single bands (100%) in females, while double and single bands were present in males. Here, 52% and 48% of males amplified with marker-3 and marker-6 had double bands. Some markers, such as marker-3 and marker-6, were 100% consistent with phenotypic sexes of all females from the four different populations. Maker-2 was 100% consistent with the phenotypic sex of males and females in only the Donghai Dao population. The markers’ consistency levels varied within and across populations (Table 1 and Table 2), indicating an incomplete sex linkage and a possible evolutionary divergence of *Dmrt2a* in spotted scat. 

**Figure 5:**
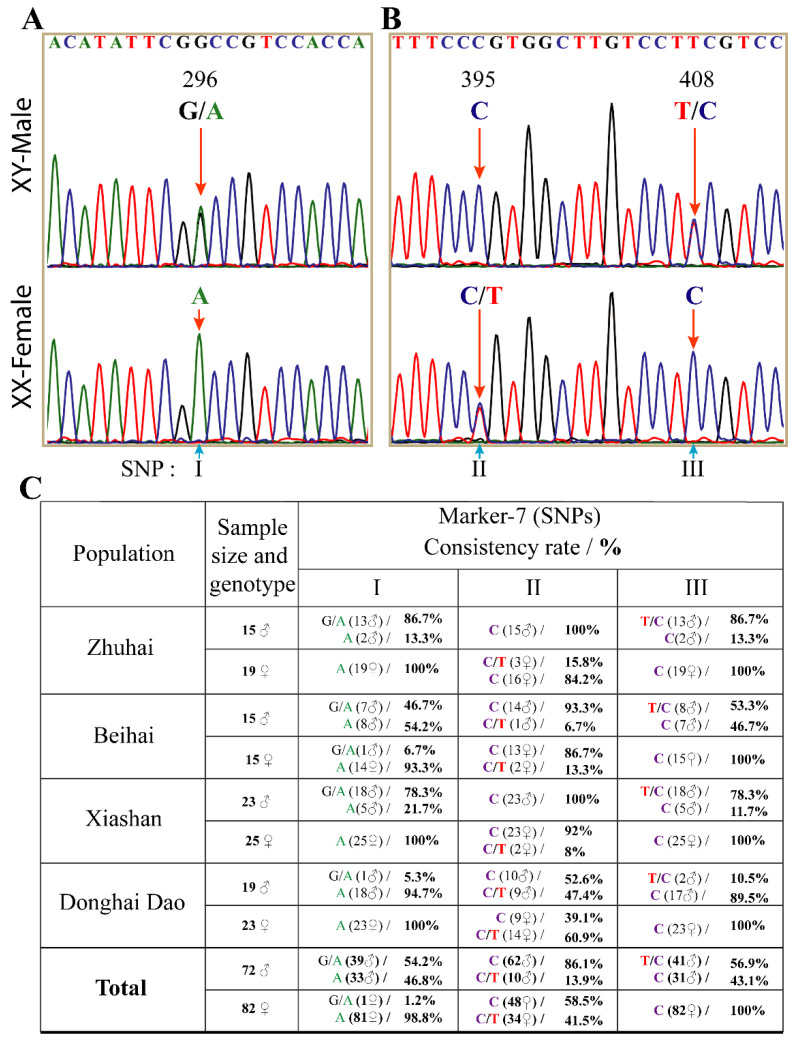
Three sex-linked SNPs in spotted scat *Dmrt2a.* SNPs in (**A**) exon2 (I) and (**B**) intron2, (II and III). (**C**) Consistency rates of the three SNPs with the genetic sex in different populations. Most males are heterozygous at I (G/A) and III (T/C) and homozygous at II (**C**), whereas females are homozygous at I (**A**) and III (**C**) and heterozygous at II (C/T).

Consistently, marker-7 spanning the three SNPs, named I on exon 2 and II and III on intron 2, also showed different concordance rates within populations (Figure 5). The 500 bp Sanger sequence results of marker-7 revealed heterozygosity (G/A) and (T/C) in XY-males and homozygous (A) and (C) in XX-females at positions I (296) and III (408), respectively. Conversely, at position II (395), XY-male was homozygous while XX-female was heterozygous. Except in one female tail fish from Beihai, which showed heterozygosity (G/A) at position I (296), all females from other populations were homozygous (A) 100%. The highest and lowest population with male heterozygosity rates at positions I and III were Zhuhai (86.7% and 86.7%) and Donghai Dao (5.3% and 10.5%), respectively (Figure 5C). On the other hand, 100% of all XY-males from Zhuhai and Xiashan were homozygous (C) at position II (395), with the least homozygosity (52.6%) observed in the Donghai Dao population. At position II, the XX-female heterozygosity (C/T) rate ranged from 93.3 in Beihai to 52.6% in Donghai Dao. Together, these markers suggest that *Dmrt2a* is incompletely sex-linked.

### 3.4. Dmrt2a Is Highly Expressed in Spotted Scat Testes

**Figure 6:**
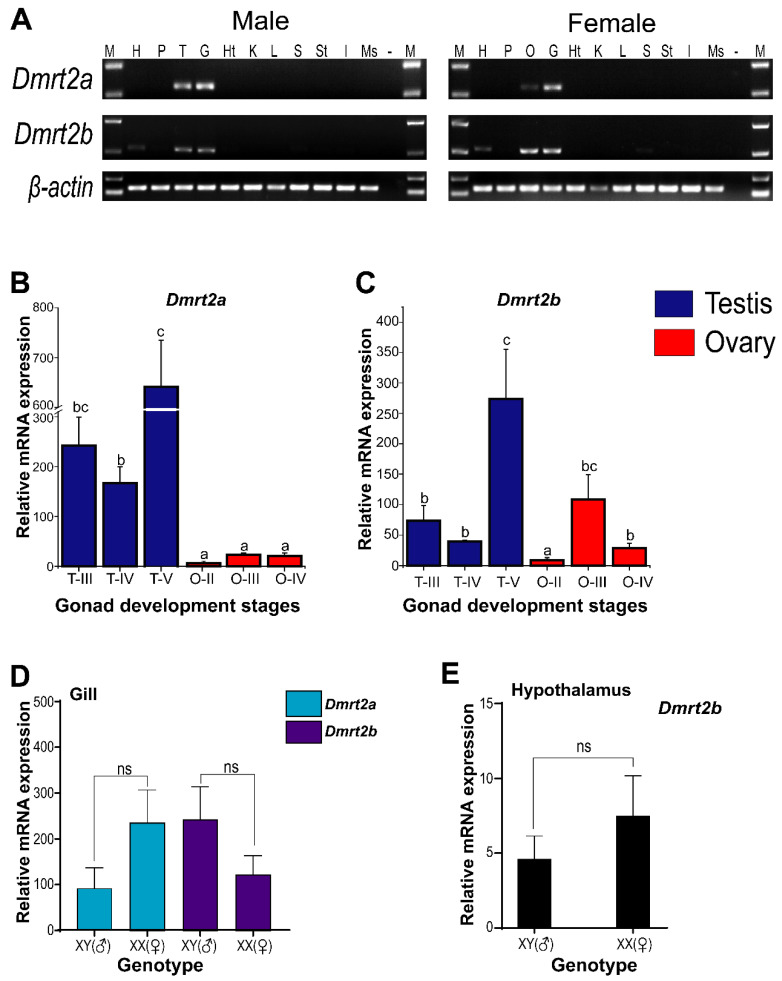
The mRNA expression levels of *Dmrt2a* and *Dmrt2b* from different tissues of adult spotted scat by PCR and qPCR. (**A**) Tissue distribution of *Dmrt2a* and *Dmrt2b. β-actin* was used as an internal control. M, DNA marker; H, hypothalamus; P, pituitary; T, testis; O, ovary; G, gill; Ht, heart; K, kidney; L, liver, S, spleen; St, stomach; I, intestine; Ms, muscle; −, negative control. Gene expression patterns of spotted scat *Dmrt2a* and *Dmrt2b* mRNA in gonads at different development stages (**B**,**C**). Gene expression patterns of spotted scat *Dmrt2a* and *Dmrt2b* mRNA in the (**D**) gills and (**E**) hypothalamus. Here, *β-actin* was used as an internal control for gene normalization, and the 2^−ΔΔCt^ method was used in gene expression calculation. The data are expressed as the means ± standard deviations of at least triplicates. Different letters indicate means with significant differences (*p* < 0.05), and ns indicate no statistically significant difference (*p* > 0.05). “T” and “O” indicate testis and ovary, respectively. The roman numerals II, III, IV, and V represent gonad development stages (stage 2, 3, 4, and 5, respectively). XY (♂) and XX (♀) represent male and female, respectively.

The tissue distribution of *Dmrt2a* and *Dmrt2b* mRNA revealed their existence in the gonads and gills of male and female spotted scat. In addition, *Dmrt2b* exists moderately in the hypothalamus and is very weak in the spleen of both males and females (Figure 6A).

The RT-qPCR gene expression in gonads at different development stages of the testes and ovaries indicated that *Dmrt2a* is expressed at significantly higher levels in the testes than in the ovaries (*p* < 0.05) (Figure 6B), while *Dmrt2b* is more highly expressed in late-stage testes than ovaries (Figure 6C). In the ovaries, *Dmrt2a* is expressed similarly in all stages (stages II, III, and IV), whereas *Dmrt2b* is significantly expressed at the middle stage (stage III). In the gills and hypothalamus of male and female spotted scat, *Dmrt2a* and *Dmrt2b* did not exhibit significantly biased expression (Figure 6D,E). Similarly, *Dmrt2b* was almost equally expressed in the hypothalamus of both males and females (Figure 6D). Together, *Dmrt2a* is more highly expressed during testes development and less so in the ovaries.

### 3.5. The Expression of Dmrt2a in Spotted Scat Gonads

**Figure 7:**
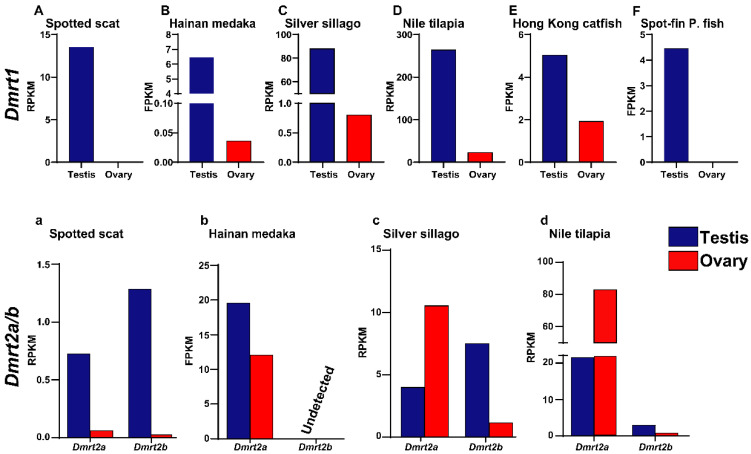
Gene expression in RPKM/FPKM obtained from gonad transcriptome data of (**A**,**a**) spotted scat [36], (**B**,**b**) Hainan medaka [38], (**C**,**c**) silver sillago [39], (**D**,**d**) Nile tilapia [40], (**E**) Hong Kong catfish [41], and (**F**) spot-fine porcupine fish [42]. *Dmrt1* is consistently expressed at higher levels in all testes. *Dmrt2a* is expressed at higher levels in the testes of spotted scat and Hainan medaka and the ovaries of tilapia and silver sillago. *Dmrt2b*, on the other hand, is expressed at higher levels in the testes of spotted scat and tilapia. *Dmrt2a* and *Dmrt2b* were not detectable in the transcriptome data for Hong Kong catfish and Spot-fin porcupine fish.

Gonadal transcriptome analysis of *Dmrt1*, *Dmrt2a*, and *Dmrt2b* from spotted scat, Hainan medaka (*Oryzias curvinotus*), silver sillago (*Sillago sihama*), Nile tilapia (*Oreochromis niloticus*), Hong Kong catfish (*Clarias fuscus*), and spot-fin porcupine fish (*Diodon hystrix*) was performed to determine their expression patterns. The results show that *Dmrt1* consistently expressed higher levels in the testes than in the ovaries (Figure 7A–F). *Dmrt2a* was highly expressed in the testes of spotted scat, consistent with the results from the qPCR test. Additionally, *Dmrt2a* was expressed highly in the testes of Hainan medaka and ovaries of silver sillago and tilapia (Figure 7a,b). Interestingly, while *Dmrt2b* was expressed highly in spotted scat, silver sillago, and Nile tilapia testes, it was not detectable in Hainan medaka (Figure 7a–d). The higher expression of *Dmrt2a* in the testes of Hainan medaka might have been influenced by the undetectable *Dmrt2b.* On the contrary, neither *Dmrt2a* and *Dmrt2b* transcripts were detectable in Hong Kong catfish and spot-fine porcupine fish annotations.

*Dmrt2a* ORF was again blasted in a different RNA-seq data of 6 male and female spotted scat samples. The expression values of *Dmrt2a* transcript in reads and FPKM were extracted (Figure S3A,i). Although the expression of *Dmrt2a* seems low, it was higher in males than in females (Figure S3A,ii), similar to Figure 7a. The results show that *Dmrt2a* expression differs even within males. Similarly, the proportion of G/A sub-types of the SNP on exon 2 (SNP I) varied within males, with the proportions of G allele (63.4%) being higher than the A allele (36.6%) (Figure S3B), while either G or A was not present in some males. The A/G sub-types are variant sequences in males and females. Therefore, the G and A sub-types occur at different rates in males and females and might represent differential expression even within males.

## 4. Discussion

The whole-genome sequencing showed that *Dmrt2a*, but not *Dmrt2b*, is close to the candidate sex determination gene (*Dmrt1*) in spotted scat. Hence, *Dmrt2a* is a good model for demonstrating the gene sequence differentiation and sexually biased expression genes in the young sex chromosome.

### 4.1. The Spotted Scat and the Incomplete Sex-Linked Dmrt2a Gene Are Excellent Models to Study the Evolution of Sex Chromosomes and the Differentiation of Sex-Linked Genes

The evolution of sex chromosomes suggests that recombination suppression leads to the degeneration of the heterogametic chromosome, such as the Y chromosome in mammals and the W chromosome in most birds. The sex chromosomes of mammals and birds are highly differentiated, with a very long evolutionary process. The mammalian Y chromosome has a long evolutionary history of more than 150 million years, whereas the W chromosome arose approximately 140 million years ago in the bird ancestor [43,44,45]. Fishes have both XY and ZW sex chromosomes systems, with ZW being the most differentiated [46]. The spotbanded scat (*Selenotoca multifasciata*) is closely related to spotted scat and diverged more than 13 million years ago, sharing the same candidate sex determination gene (*Dmrt1*) ([3] unpublished data). This implies that the spotted scat sex chromosome may be young and an excellent model to study the evolution of sex chromosomes in vertebrates.

Markers designed on the *Dmrt2a* transcript indicate that *Dmrt2a* is partially sex-linked in spotted scat. The spotted scat *Dmrt2a* exhibits varying sequence variations with several insertions and deletions (indels) and single-nucleotide polymorphisms (SNPs) between males and females. However, the sequence of *Dmrt2a* is functional, unlike *Dmrt3△-Y* and *Dmrt1b*, although differences might be present in the promoter region. XX spotted scat lack a normal *Dmrt1*, yet growth is not affected, indicating that *Dmrt1* is not essential for survival. Consistently, *Dmrt1* −/− zebrafish grow like normal female [47]. Additionally, *Dmrt3* gene knockouts studies showed that it is not critical for mouse survival [48]. Therefore, the dysfunctional mutation on the sex-linked region will most probably occur on genes whose functions are not essential for the survival of both sexes or the sexual maintenance of one sex. Hence, the spotted scat *Dmrt2a* might have a vital role in the developmental process. Additionally, the conservation differences between *Dmrt1*, *Dmrt3*, and *Dmrt2a* might be due to the effect of the gene dosage, since correct dosage is required for the proper functioning of the gene, or specialization of the truncated copies of *Dmrt1* and *Dmrt3* might be necessary for regulating normal gene copies [49,50].

Markers spanning the indels and SNPs tested on four populations indicated that the mutations are sex-linked at different rates, with males characterized by high heterogeneity. The mutations may be variably sex-linked in different populations because of the genetic variation required for evolutionary novelty and adaptation [51]. Interestingly, most of these mutations are found in the intronic regions, which is plausibly why there was no effect on the gene’s translation. Additionally, the high polymorphism in males can be explained in alternative ways as follows: (1) all indels originated from *Dmrt2a* on the Y chromosome and rare crossing-over transferred the mutated alleles onto the X chromosome; (2) the *Dmrt2a* introns might contain regulatory elements that in females play a more critical role in viability or reproduction than in males. The expression of *Dmrt2a* in the gonads at the early stages of development should be investigated. Additionally, future research is required to identify the regulatory regions.

It is worth noting that *Dmrt3* is positioned closer to the candidate sex determination gene (*Dmrt1*) (Figure 1 and Table S3), likely resulting in its quick differentiation. On the other hand, *Dmrt2a* is farther away from *Dmrt1*, which means more time may be needed for the differentiation to occur. Although in zebrafish *Dmrt2a* mutation is not lethal in experimental conditions [52], we cannot rule out the possibility of *Dmrt2a* exerting an essential function in developmental processes or adaptable characteristics in the natural environment. The critical function of *Dmrt2a* might restrict the accumulation of the dysfunctional mutation on its sequences. As such, the spotted scat *Dmrt2a* sequence variation will be monitored in the future.

### 4.2. Reason for the Sexually Dimorphic Expression of Dmrt2 in the Gonads of Spotted Scat

Gene expression studies have gained research interest in recent years because of the importance of understanding the changes in biological processes. Gene expression can provide a snapshot of actively expressed genes and transcripts in developmental pathways or responses or adaptions to new conditions. In recent years, most researchers have been interested in genes that exhibit sexually dimorphic expression patterns. Fortunately, technological advancements such as transcriptome analysis have made identifying gonadal sexual biased genes easy. Numerous sex-biased genes have since been identified in many other species [41,42,53,54,55], including Nile tilapia [40] and spotted scat [36]. Meanwhile, most of these studies have focused on gonadal gene expression, with little or no interest in the possible mechanism leading to sex difference. Ideally, sex-linked genes might show sexually biased expression [3,36,56].

The spotted scat *Dmrt2a* exhibits sex-biased expression. However, the sexually dimorphic expression of *Dmrt2a* might not necessarily be due to it being sex-linked. Autosomal *Dmrt2a* of Hainan medaka, silver sillago, and Nile tilapia also showed sexually dimorphic expressions. Several factors may affect gene expression patterns. For instance, transcriptional regulation can affect the pattern of gene expression [57]. In addition, epigenetic changes in the actual DNA sequence can also affect gene expression. In fighting fish, *Dmrt1* shows male-biased gonadal expression, while the reduced expression in female is caused by epigenetic changes [58]. This epigenetic change is induced by the *Drbx1* gene at the promoter region of *Dmrt1* [58]. The spotted scat *Drmt2a* promotor region also has several polymorphisms. It is unclear whether the sex-biased expression of the spotted scat *Dmrt2a* is caused by the polymorphism at the promoter region or DNA methylation. Additionally, upstream genes are promoter sequences, natural regulators of gene expression, and major gene expression regulatory elements [59]. Therefore, further studies such as epigenetic modification, ATAC sequencing, and X and Y sequence variation analysis are needed to elucidate the regulators of *Dmrt2a* expression in spotted scat.

## 5. Conclusions

Herein, the spotted scat chromosome is not old, making it an excellent model to study the evolution of sex chromosomes in teleosts. In spotted scat, *Dmrt2a* is positioned close to the sex-specific *Dmrt3* and *Dmrt1* with the three genes spanning across an estimated distance of 40 kb on the sex chromosome. The *Dmrt2a* is highly mutated at the non-coding region, with males exhibiting high heterogeneity. The results infer that *Dmrt2a* is partially sex-linked in spotted scat. *Dmrt2a* is functional and might regulate other developmental processes, or it is yet to be differentiated in spotted scat. Therefore, *Dmrt2a* is an excellent model for studying sex-linked gene differentiation during the evolution of vertebrates. The expression patterns of *Dmrt2a* in gonads fluctuate among different species, suggesting different functions in different species. Hence, studies to elucidate the function of *Dmrt2a* in spotted scat will bring to bear its role in developmental processes.

## Figures and Tables

**Table 1:** Comparisons of marker-2 and marker-3 with genetic sex of spotted scat samples from different populations.

Population	Sample Size and Genotype	Marker-2/Indel2-8 bp (Intron1)		Marker-3/Indel3-12 bp (Intron3)	
		Consistency	Average Concordance Rate (%)	Consistency	Average Concordance Rate (%)
Donghai Dao				a/b (1 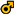 )	5.3
	19 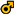	a/b (19 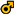 )	100	b (18 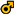 )	94.7
	25 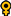	a/b (25 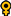 )	100	b (25 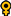 )	100
Beihai	17 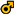	a (10 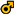 ) a/b (7 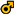 )	58.8 41.2	a (1 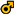 )	5.9
				a/b (7 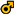 )	41.2
				b (9 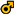 )	52.9
	13 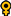	a (5 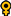 )	38.5		
		a/b (8 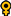 )	61.5	b (13 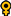 )	100
Xiashan	24 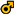	a (17 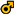 )	70.8	a/b (18 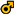 )	75
		a/b (7 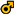 )	29.2	b (6 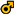 )	25
	24 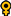	a (6 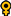 )	25	b (24 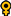 )	100
		a/b (11 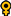 )	45.8		
		b (7 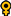 )	29.2		
Zhuhai	15 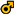	a (9 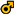 )	60	a/b (13 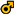 )	86.7
		a/b (6 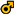 )	40	b (2 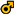 )	13.3
	19 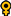	a (1 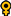 )	5.3	b (19 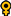 )	100
		a/b (9 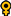 )	47.4		
		b (9 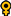 )	47.4		
TOTAL	75 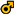	a (36 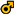 ) a/b (39 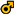 )	48 52	a (1 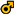 )	1.3
				a/b (39 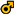 )	52
				b (35 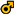 )	46.7
	81 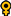	a (12 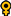 )	14.8	b (81 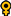 )	100
		a/b (53 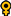 )	65.4		
		b (16 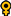 )	19.8		

Note: For marker-2, bands “a”, “a/b” indicate 147 bp and (147 and 139 bp) bands, respectively. For marker-3, bands “a” “a/b” and “b” indicate a 160 bp, (160 and 148 bp) and 148 bp bands, respectively. The “a” and “b” represent shorter and longer bands.

**Table 2:** Comparison of marker-4 and marker-6 with genetic sex of spotted scat from different populations.

Population	Sample Size and Genotype	Marker-4/Indel4-99 bp (Intron1)		Sample Size and Genotype	Marker-6/Del6-14 bp (Intron2)	
		Consistency	Average Concordance Rate (%)		Consistency	Average Concordance Rate (%)
Donghai Dao	23 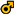	a (13 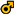 )	56.5		a (3 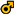 )	14.3
		a/b (10 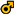 )	43.5	21 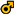	a/b (18 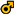 )	85.7
	25 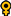	a/b (2 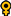 )	8		a (25 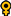 )	100
		b (23 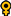 )	92	25 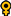		
Beihai	17 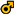	a (9 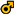 )	52.9		a (7 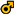 )	46.7
		a/b (8 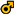 )	47.1	15 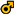	a/b (8 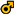 )	53.3
	13 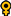	a (2 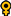 )	15.4	15 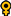	a (15 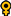 )	100
		a/b (6 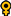 )	46.2			
		b (5 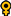 )	48.5			
Xiashan	24 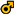	a (9 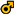 )	37.5		a (16 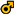 )	64
		a/b (15 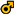 )	62.5	25 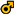	a/b (9 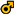 )	36
	24 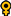	a (4 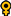 )	16.7	30 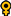	a (30 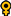 )	100
		a/b (8 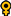 )	33.3			
		b (12 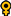 )	50			
Zhuhai	16 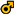	a (4 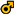 )	25		a (14 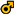 )	87.5
		a/b (12 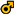 )	75	16 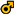	a/b (2 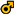 )	12.5
	22 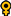	a (1 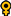 )	4.5	23 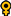	a (23 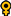 )	100
		a/b (2 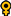 )	9.1			
		b (19 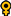 )	86.4			
TOTAL	80 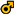	a (35 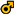 )	43.7		a (40 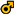 )	51.9
		a/b (45 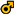 )	56.3	77 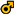	a/b (37 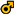 )	48.1
	84 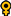	a (7 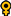 )	8.3	93 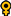	a (93 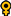 )	100
		a/b (18 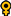 )	21.4			
		b (59 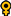 )	70.2			

Note: For marker-4, bands “a”, “a/b” and “b” indicate 543 bp, (453 and 444 bp) and 444 bp bands, respectively. For marker-6, bands “a” and “a/b” indicate 158 bp, and (158 and 144 bp) bands, respectively. The “a” and “b” inscriptions are shown in Figure 4.

## Data Availability

Not applicable.

## Supplementary Materials

The following supporting information can be downloaded at: https://www.mdpi.com/article/10.3390/ani12050613/s1. Figure S1: Nucleotide and deduced amino acid (aa) sequences of spotted scat *Dmrt2a* (A) and *Dmrt2b* (B). Nucleotides and amino acid (aa) sequences are numbered to the right. Rectangle indicates the conserved DM domain region. The putative nuclear localization signals (NLSs) are highlighted in grey. Downward and upward arrows indicate start and stop (*) codons and conserved cysteine and histidine residues, respectively. Figure S2: *Dmrt2a* sequence analysis in spotted scat. (AI) At most 14 bp indels in intron 1 of male and female sequences (AII), PAGE gel with primers spanning the indel region (marker-1) did not show distinguishable bands in males and females. (BI) At most 10 bp indels in intron 1 of male and female sequences (BII), PAGE gel with primers spanning the indel region (marker-5) did not show distinguishable bands between males and females. At least 16 TG single sequence repeats (SSR-1 and SSR-2) preceded indel 1 and indel 5. The indel 1 and the indel 5 are positioned first and last on intron1. Table S1: Primer sequences used in this study. Table S2: NCBI and Ensembl sequence IDs of Dmrt proteins used in this study. Figure S3. A) *Dmrt2a* transcript expression by transcriptome analysis. i) *Dmrt2a* FPKM and reads values from 6 male and 6 female transcriptome data. ii) Graph of average FPKM values and reads numbers from the 6 males and females in "i" above. B) The proportion of G and A sub-types in six XY-males (male 1 - 6). The SNP region (SNP: I) on the Exon 2 of *Dmrt2a* transcript was used as a querry sequence to blast the row RNA-seq data of 6 XY-males using blastX. The proportion of clean reads with either G or A were determined. Male 1 has very low reads, and the SNP site was not found. Male 2 and (4 and 5) did not have G and A alleles, respectively. On average, the proportion of the G allele is more (63.4%) than A (36.6%).
