# Supplementary material for: High Polymorphism in the Dmrt2a Gene Is Incompletely Sex-Linked in Spotted Scat, Scatophagus argus
Source: Animals (Basel). 2022 Feb 28;12(5):613. doi: 10.3390/ani12050613 (PMC8909180; doi:10.3390/ani12050613)
Supplement: Supplementary file 1 [file animals-12-00613-s001.zip › animals-1581323-supplementary.pdf]

Supplementary Data

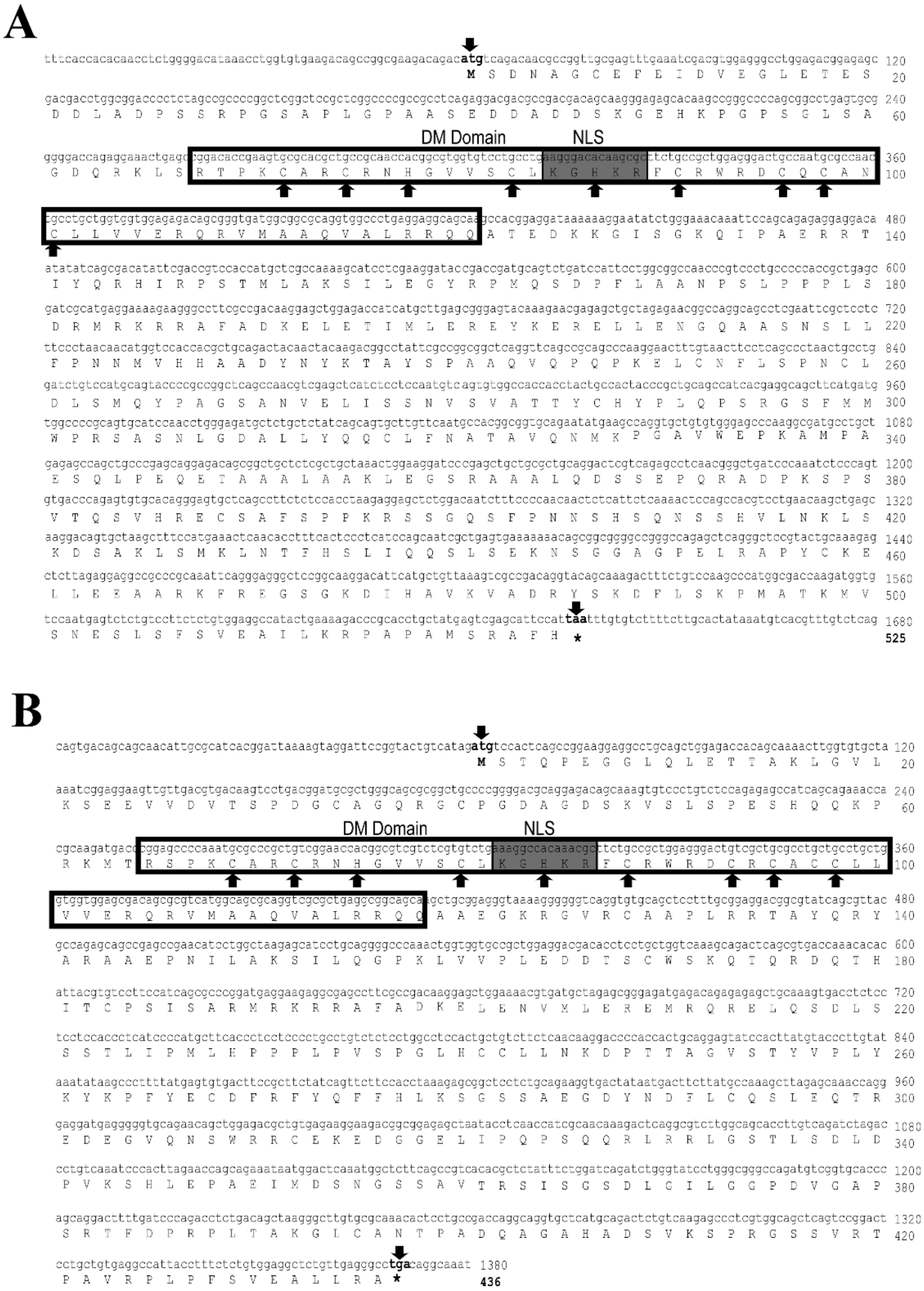

Figure S1. Nucleotide and deduced amino acid (aa) sequences of Spotted scat Dmrt2a (A) and Dmrt2b (B). Nucleotides and amino acid (aa) sequences are numbered to the right. Rectangle indicates the conserved DM domain region. The Putative nuclear localization signals (NLSs) are highlighted in grey. Down and up arrows indicate (start and stop (*) codon) and (conserved cysteine and histidine) residues, respectively.

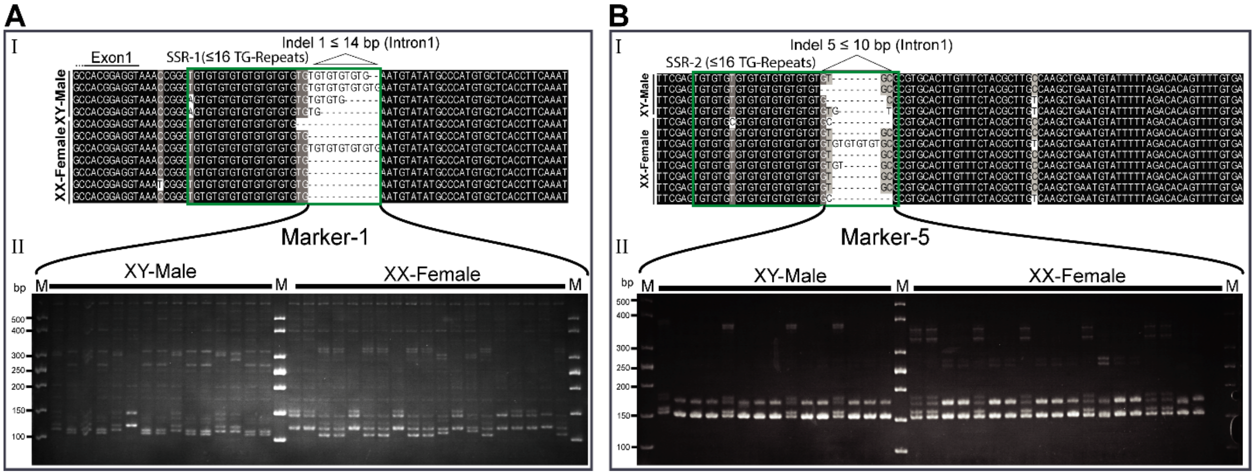

Figure S2. *Dmrt2a* sequence analysis in spotted scat. **A, I**) At most 14 bp indel in intron 1 of male and female sequence **A, II**) PAGE gel with primers spanning the indel region (Marker-1) did not show distinguishable bands in males and female. **B, I**) At most 10 bp indel in intron 1 of male and female sequence. **B, II**) PAGE gel with primers spanning the indel region (Marker-5) did not show distinguishable bands between males and female. At lease 16 TG single sequence repeats (SSR-1 and SSR-2) preceeded indel 1 and indel 5. The indel 1 and the indel 5 are positioned first and last on intron 1.

A

i

Expression	Sex	Fish 1	Fish 2	Fish 3	Fish 4	Fish 5	Fish 6
FPKM	Male	0.081595	1.887699	0.671748	0.567301	0.155794	1.408063
	Female	0	0.393898	0.259623	0.088912	0	0.062524
Reads	Male	9	101	53	33	10	95
	Female	0	28	18	6	0	5

ii

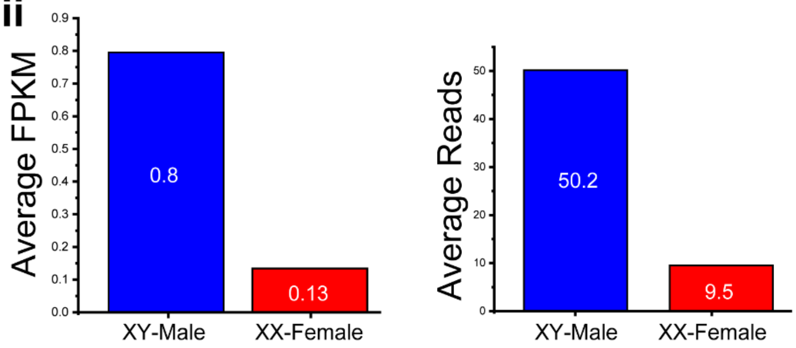

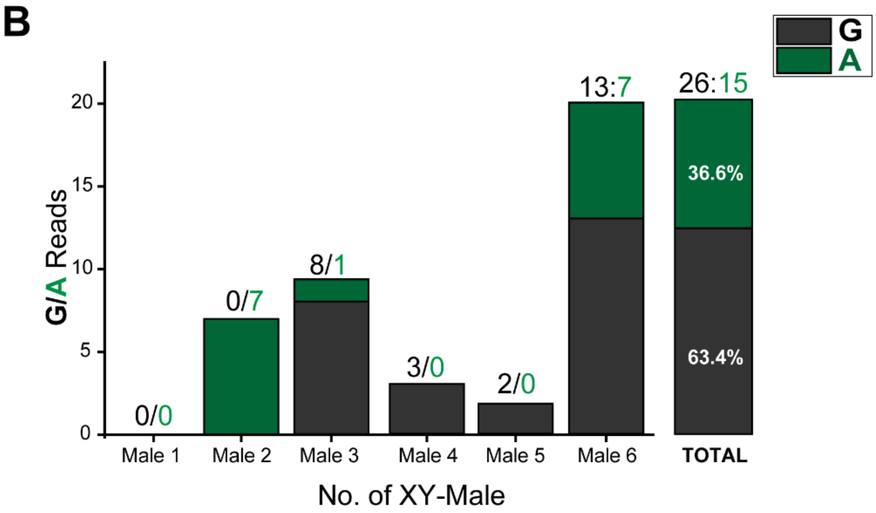

Figure S3. A) *Dmrt2a* transcript expression by transcriptome analysis. i) *Dmrt2a* FPKM and reads values from 6 male and 6 female transcriptome data. ii) Graph of average FPKM values and reads numbers from the 6 males and females in "i" above. B) The proportion of G and A sub-types in six XY-males (male 1 - 6). The SNP region (SNP: I) on the Exon 2 of *Dmrt2a* transcript was used as a querry sequence to blast the row RNA-seq data of 6 XY-males using blastX. The proportion of clean reads with either G or A were determined. Male 1 has very low reads, and the SNP site was not found. Male 2 and (4 and 5) did not have G and A alleles, respectively. On average, the proportion of the G allele is more (63.4%) than A (36.6%).

Table S1. Primer sequences used in this study

Primer name	Forward 5′ – 3′	Reverse 5′ – 3′	Product size bp
	**Cloning (Open Reading Frame, ORF)**		
Dmrt2a	GCTAATTCAATTCTTGTCCTGGAGTCTG	GACAAACGTGACATTTATAGTGCAAG	1786
Dmrt2b	GCTCAGTGACAGCAGCAACATTG	GCACTTCATCACTTTCCATTTGCCTG	1400
	**Cloning gDNA**		
Dmrt2a	GTTGCGAGTTTGAAATCGACGTG	ACAAACGTGACATTTATAGTGC	4042
	**Tissue distribution / Gene expression**		
Dmrt2a	GACAACGCCGGTTGCGAGTT	TCCACCACCAGCAGGCAGTT	311
Dmrt2b	TGTCCACTCAGCCGGAAGGA	CTCCAGCGGCAGAAGCGTTT	271
β-actin	GAGAGGTTCCGTTGCCCAGAG	CAGACAGCACAGTGTTGGCGT	
	**Markers**		
Marker-1	TGGAGAGACAGCGGGTGATG	TGAAGGTGAGCACATGGGCATA	129
Marker-2	TGTATATGCCCATGTGCTCACCTTC	GGGAAACAGCACTGACTGACCCGAG	147, 139
Marker-3	AGATCCTGAGCAGCAGATATTGTAGG	TGAAACTAAAAATAGTACTGGTTTCTTAC	160, 148
Marker-4	GACCATAACGAAGGCGAACACATT	AAAGTGGGACAGTGAGTCAAATGG	543, 444
Marker-5	ATGGTACTGTATGGATGTCTTTCTG	AACTAAGCCTGACTGCATTACTTCA	158
Marker-6	CGTCCCAGTTTAACAGCGATA	TGTTGTGACAGTGCTGGACATG	158, 144
Marker-7 (SNP)	AGTTGTGCTAGACCTCCATCTCATG	CTAATGCCACCAAGTCAAGTATGTT	500

Table S2. NSBI and Ensembl sequence IDs of Dmrt proteins used in this study

Species name	Accession Number
**Dmrt2/a**	
Zebrafish, *Danio rerio*	NM_130952.1
Nile tilapia, *Oreochromis niloticus*	NM_001279767.1
Fugu, *Takifugu rubripes*	ENSTRUG00000015666.3
Medaka, *Oryzias latipes*	ENSORLG00000022893.1
Spotted gar, *Lepisosteus oculatus*	XM_006627124.2
Coelacanth, *Latimeria chalumnae*	XM_014491555.1
Stickleback, *Gasterosteus aculeatus*	ENSGACG00000014508.1
Frog, *Xenopus laevis*	NM_001096256.1
Lizard, *Anolis carolinensis*	XM_008103322.2
Chicken, *Gallus gallus*	XR_005843092.1
Mouse, *Mus musculus*	NM_145831.3
Human, *Homo sapiens*	NC_000009.12
Spotted scat, *Scatophagus argus*	MG765301.1
**Dmrt2b**	
Zebrafish, *Danio rerio*	NM_001079976.1
Nile tilapia, *Oreochromis niloticus*	NM_001279473.1
Fugu, *Takifugu rubripes*	ENSTRUG00000018484.3
Medaka, *Oryzias latipes*	ENSORLG00000025087.1
Spotted gar, *Lepisosteus oculatus*	XM_006634912.2
Stickleback, *Gasterosteus aculeatus*	ENSGACG00000006816.1
Spotted scat, *Scatophagus argus*	MG765302.1
**Dmrt5**	
Spotted scat, *Scatophagus argus*	MG765305.1
